# Supplementary material for: Free Energy Principle in Human Postural Control System: Skin Stretch Feedback Reduces the Entropy
Source: Sci Rep. 2019 Nov 14;9:16870. doi: 10.1038/s41598-019-53028-1 (PMC6856340; doi:10.1038/s41598-019-53028-1)
Supplement: Supplementary file 1 — Supplementary information [file 41598_2019_53028_MOESM1_ESM.pdf]

Supplementary Information

Title: Free Energy Principle in Human Postural Control System: Skin Stretch Feedback Reduces the Entropy

Authors: Pilwon Hur, Yi-Tsen Pan, and Christian DeBuys

Appendix

The following shows that minimizing the free energy of the self-organizing biological system is implicitly equivalent to minimizing the surprise and entropy. Let S be the set of the sensory states, and R be the set of the internal states. For $s \in S, \mu \in R,$ and m a generative model, or forward model, a surprise is defined as $-\log p(s|m)$ which is a self-information and describes the accuracy of the model[1]. Kullback-Leibler divergence term appears in the free energy to resolve the intractability of the marginalization in computing the posterior density by introducing variational (or, approximate) density. Then, we find that the free energy is the upper bound of the surprise which is the log of negative self-evidence. In other words, the surprise is the lower bound of the free energy.

$$F(s,\mu) = -\log p(s|m) + D_{KL}[q|p] \geq -\log p(s|m)$$

where $D_{KL}[\cdot \mid \cdot]$ is the Kullback-Leibler divergence between the posterior density and the variational density and is always nonnegative. Kullback-Leibler divergence becomes zero when the two densities are identical.

The transition matrix, P, of the postural sway is irreducible by the construction[2] and aperiodic by its nature[3]. Note that the transition matrix, P, is an approximation of the Perron-Frobenius operator. Thus, P has the unique invariant density π and the process of the postural control system can be assumed to be ergodic[2,4,5]. Therefore, the long-term average of the free energy is the upper bound of the entropy of sensory states as shown below.

$$\lim_{T\to\infty} \frac{1}{T}\int_0^T F\big(s(t),\mu(t)\big)dt \geq \lim_{T\to\infty}\frac{1}{T}\int_0^T -\log p(s|m)\, dt = \int_0^T -p(s|m)\log p(s|m)\, ds = H[p(s|m)]$$

References

1. Jones, D. *Elementary information theory* (Oxford University Press, Oxford, UK, 1979).
2. Hur, P., Shorter, K., Mehta, P. & Hsiao-Wecksler, E. Invariant density analysis: Modeling and analysis of the postural control system using markov chains. *IEEE Trans Biomed Eng* 59, 1094–1100 (2012).
3. Milton J., S. G., Insperger T. Human balance control: Dead zones, intermittency, and micro-chaos. In Ohira T., U. T. (ed.) *Mathematical Approaches to Biological Systems*, chap. 1, 1–28 (Springer, Tokyo, 2015).
4. Birkhoff, G. Proof of the ergodic theorem. *Proc Natl Acad Sci* 17, 656–660 (1931).
5. Lasota, A. & Mackey, M. *Chaos, Fractals, and Noise: Stochastic Aspects of Dynamics* (Springer, New York, USA, 1994), 2nd edn.
